# Förster Resonance Energy Transfer in Metal Halide Perovskite: Current Status and Future Prospects

**DOI:** 10.1002/open.202400118

**Published:** 2024-12-04

**Authors:** Siyang Liu, Waseem Akram, Fanghao Ye, JingCheng Jin, Fangfang Niu, Shakeel Ahmed, Zhengbiao Ouyang, Shou‐Cheng Dong, Guijun Li

**Affiliations:** ^1^ Key Laboratory of Optoelectronic Devices and Systems of Ministry of Education and Guangdong Province College of Physics and Optoelectronic Engineering Shenzhen University Shenzhen, 518060 China; ^2^ State Key Laboratory of Advanced Displays and Optoelectronics Technologies Department of Electronic and Computer Engineering The Hong Kong University of Science and Technology Kowloon,999077 Hong Kong; ^3^ School of Microelectronics Shenzhen Institute of Information Technology Shenzhen, 518000 China; ^4^ WISPO Advanced Materials (Suzhou) Co., Ltd. Suzhou, 215000 China

**Keywords:** FRET, Metal halide perovskites, Energy transfer mechanism, Inorganic fluorophores, Organic fluorophores

## Abstract

Förster Resonance Energy Transfer (FRET) is a non‐radiative energy transfer process in a donor‐acceptor system and has applications in various fields, such as single‐molecule investigations, biosensor creation, and deoxyribonucleic acid (DNA) mechanics research. The investigation of FRET processes in metal halide perovskites has also attracted great attention from the community. The review aims to provide an up‐to‐date study of FRET in the context of perovskite systems. First, we discuss the fundamentals of FRET process, and then summarize the recent progress of FRET phenomenon in perovskite‐perovskite, perovskite‐inorganic fluorophores, perovskite‐organic fluorophores, and organic fluorophores‐perovskite systems. Finally, we speculate on the future prospects of roles of FRET in the implications for the overall performance of optoelectronic devices based on these systems, as well as the challenges in maximizing FRET efficiency.

## Graphical abstract

This paper provides a fundamental understanding of Förster Resonance Energy Transfer (FRET) and summaries recent progress of the exploration of FRET in the content of perovskite‐perovskite, perovskite‐inorganic, and perovskite‐organic systems. The future prospects of roles and challenges of FRET in the implications for the overall performance of perovskite‐based optoelectronic devices are also discussed.

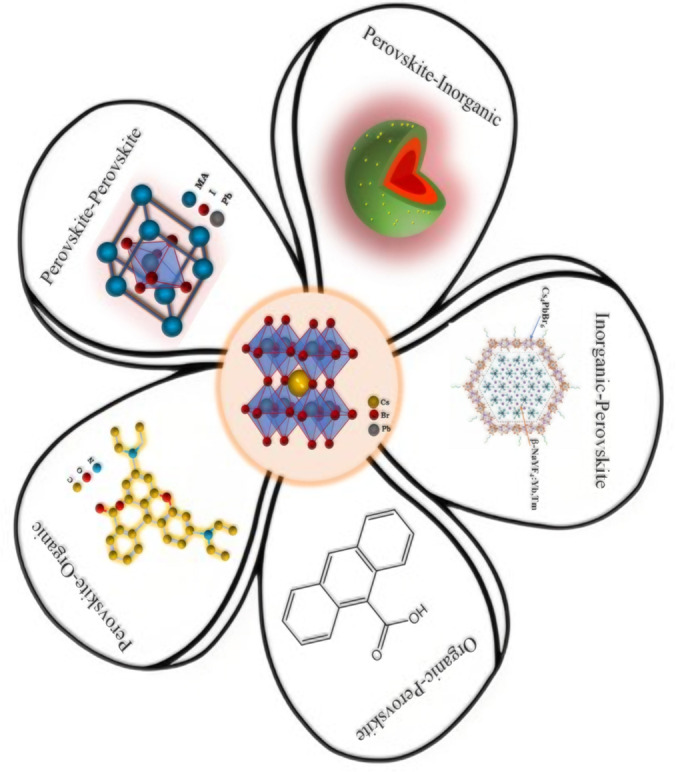

## 1 Introduction

Förster resonance energy transfer (FRET) is a nonradiative energy transfer process that involves the transfer of energy from a fluorophore in an excited electronic state (donor) to another fluorophore in the ground electronic state (acceptor) through dipole‐dipole coupling.^[1]^ FRET is commonly used to measure the distance between two positions of interest on a large molecule, typically a biological macromolecule, by attaching appropriate donor‐acceptor groups to it.^[2]^ Because FRET operates within a range of 1–10 nm that corresponds to the dimensions of macromolecules, it can provide a high level of specificity when analyzing molecular interactions.^[3]^ FRET can also be used to measure dynamic activities between two sites on a macromolecule, such as protein interactions, assuming there are no significant conformational changes during the process.^[3]^ In addition, FRET can be found in applications such as single‐molecule experiments, molecular motors, biosensors, photocatalysis,^[4]^ bioimaging,^[5]^ and deoxyribonucleic acid (DNA) mechanical movements, and is therefore often referred to as the “spectroscopic ruler” due to its convenience.^[6]^

Recently, it has been demonstrated that FRET can be utilized for optoelectronic technologies with the objective of developing lighting and solar energy–harvesting systems that are extremely efficient.^[7]^ There have been numerous applications of FRET in the field of molecular biology, including sensing, labelling, nanoscale distance measurements.^[8]^ A solid understanding of the theoretical framework within which FRET operates is vital to accurately forecast and optimize energy transfer processes in various systems.

Metal halide perovskites (MHPs) have emerged as highly promising optoelectronic materials in the field of photovoltaics (PVs), light‐emitting diodes (LEDs), photodetectors (PDs), and lasers due to their unique characteristics, including the ability to adjust their bandgap,[^9^, ^10^, ^11^, ^12^, ^13^] high absorption coefficient,[^14^, ^15^, ^16^] and cost‐effective manufacturing methods.[^17^, ^18^, ^19^, ^20^, ^21^] The investigation of FRET in perovskite‐based optoelectronics has garnered attention due to its ability to enhance device performance and functionality.[^22^, ^23^, ^24^, ^25^] For example, researchers have investigated the incorporation of FRET processes in solar cells to boost the light harvesting efficiency.[^26^, ^27^] FRET‐enabled LEDs[^28^, ^29^] have been shown to produce a wider range of light emission. FRET‐based PDs and sensors can also be used to identify a wider range of analytes more accurately and selectively.^[30]^ Even though, the exploration of FRET in the content of MHP system faces many obstacles and requires continues research in future directions. To take full advantage of FRET in this rapidly developing sector, strategies that focus on enhancing energy transfer efficiency, improving device structures, and exploring novel applications must be implemented.^[31]^ This review article aims to provide a comprehensive understanding of the mechanics of FRET, summary recent advances of FRET in MHPs, and speculate on the future prospects of roles and challenges of FRET in the exciting field of perovskite research.

## 2 Fundamentals of Förster Resonance Energy Transfer

**Figure 1:**
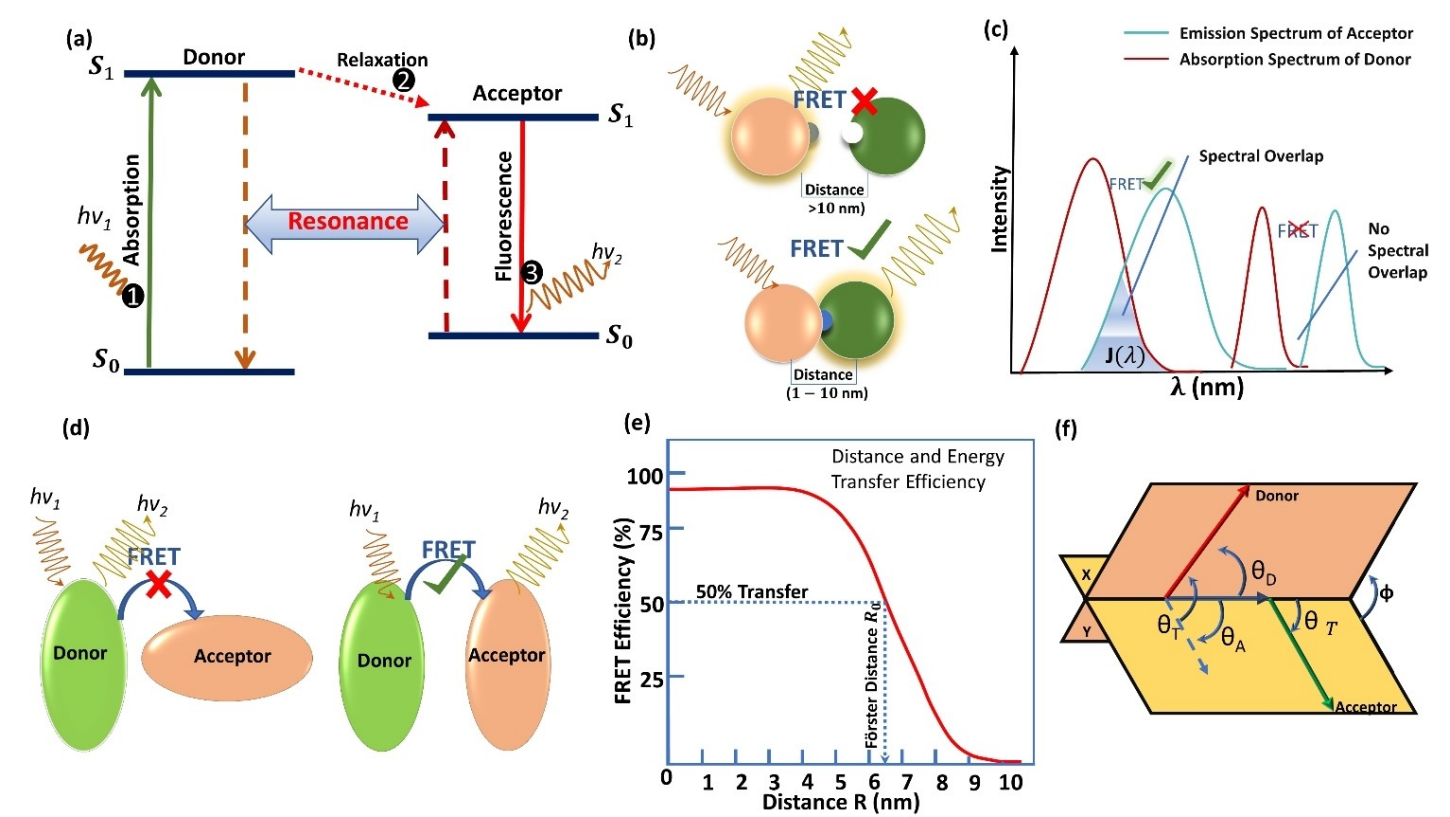
a) Schematic diagram of FRET process between donor and acceptor fluorophores. b) Representing the FRET process depending on the distance between acceptor and donor, if distance is larger than 10 nm FRET will not happen. Distance should be 1–10 nm between Donor and acceptor molecule for FRET process. c) Overlap integral *J*(λ ) for donor and acceptor fluorophores showing Energy transfer by overlapping. d) illustrate the orientation of the donor and acceptor if both are in parallel orientation then FRET will happen otherwise there will be no FRET process. e) Graphical the relationship between transfer efficiency and the distance separating the donor and acceptor is demonstrated to be exponential. The efficiency reaches 100 % as the separation distance decreases below $R_{0}$ , and conversely, drops to zero when R is greater than $R_{0}$ . At around 50 % of $R_{0},\;$ the FRET efficiency is close to its maximum, making it challenging to accurately determine shorter distances. If the donor‐acceptor distance surpasses the $R_{0}$ value by 50 %, the curve of slope becomes so gradual that longer separation distances become indistinguishable. f) The equation that determines the orientation factor $k^{2}$ is dependent on the relative position of the donor emission dipole and the acceptor absorption dipole. This equation can be expressed as follows: $k^{2}$ =(cos ${\theta \;}_{T}$ ‐3cos θD cos θA )^2^=(sin θD sin θA cos Φ‐2cos θD cos θA )^2^. Here, ${\theta \;}_{T}$ represents the angle between the emission transition dipole of the donor and the absorption transition dipole of the acceptor, while θD and θA denote the angles between these dipoles and the vector connecting the donor and acceptor. Additionally, Φ stands for the angle between the planes that contain the two transition dipoles.

The theoretical study conducted by Theodor Förster greatly advanced our understanding of the FRET mechanism. As shown in Figure 1(a), in FRET, a donor group (D) is excited by a photon and transited to the lowest excited singlet state (S_1_), as per Kasha's rule.[^7^, ^8^] If an acceptor group (A) is in close proximity to the donor, the energy released when the donor relaxes to the ground state (S_0_) and can be transferred non‐radiatively to the acceptor, not re‐exciting the donor. If there are no more quenching states, the stimulated acceptor releases a photon and returns to the ground state. The energy transfer in FRET is strongly influenced by the Coulombic interaction between the donor and acceptor, meaning that FRET is primarily a dipole‐dipole interaction rather than a direct electron transfer process.^[34]^

The energy transfer efficiency is determined by the interaction between the transition dipoles of the donor and the acceptor, which can be described by the Förster equation, given as:

(1) $$E=\frac{1}{1+{\left(\frac{R}{R_{0}}\right)}^{6}}$$

Where, E is the energy transfer efficiency ranging from 0 to 1.

R is the distance between the donor and acceptor.

$R_{0}$ is the Förster radius, representing the distance at which the FRET efficiency is 50 %. There is a reciprocal correlation between the effectiveness of FRET and the sixth power of the distance that separates the donor and the acceptor. According to Equation (1), FRET is determined by the distance between the donor and acceptor. In most cases, FRET only occurs in a distance range of 1–10 nm. If the distance is larger than 10 nm, there is no FRET, as shown in Figure 1(b).

R_0_ can be calculated by using the following Equation 2:

(2) $$R_{0}^{6}=\frac{9\cdot ln\left(10\right)\cdot k^{2}\cdot Q_{D}\cdot J\left(\lambda \right)}{128\pi^{5}\cdot n^{4}\cdot \varphi_{D}\;}$$

Here, $Q_{D}$ is the fluorescence quantum yield of the donor in the absence of the acceptor.$\;k^{2}$ is the dipole orientation factor. n is the refractive index of the medium.$\;J\left(\lambda \right)$ is the spectral overlap. $\varphi_{D}$ is the quantum yield of the exited state of the donor.

From Equation (2), the energy transfer efficiency is further influenced by factors of spectral overlap (Figure 1(c)), orientation of dipoles (Figure 1(d)), and molecular interactions (Figure 1(e)).

The maximum amount of energy may be transferred by FRET process when the transition dipoles are aligned and the distance of the dipoles ranging from 1 to 10 nm, while the Dexter energy transfer is limited to shorter distances (up to 1–2 nm) and takes place via electron exchange. Additionally, the spectral overlap that exists between the emission spectrum of the donor and the absorption spectrum of the acceptor is also a critical factor in determining the efficacy of the FRET. FRET only occurs in the case of spectral overlap. The concept of the overlap integral $J\left(\lambda \right)$ is given by Equation 3:

(3) $$J\left(\lambda \right)=\int_{0}^{\infty }F_{D}\left(\lambda \right)\epsilon_{A}\left(\lambda \right)\lambda^{4}d\lambda$$

Here, $F_{D}\left(\lambda \right)$ represents the emission spectrum of the donor, whereas $\epsilon_{A}$ is the molar absorption coefficient of the acceptor. λ refers to the wavelength.

The overlap integral quantifies the extent to which the emission spectrum of the donor overlaps with the absorption spectrum of the acceptor. This represents the degree of overlap between the two spectra influences the probability of energy transfer. Förster also established useful terms for the orientation component was shown in Figure 1(f),$\;k^{2}$ , and incorporated the influence of the refractive index, which impacts all electrical interactions in condensed media at elevated optical frequencies. Because of this distance dependency, FRET is extremely sensitive to changes in the molecule separation, which enables exact measurements of molecular interactions at the nanoscale.[^4^, ^5^] Quantum confinement of the acceptor fundamentally alters the distance dependency of FRET by affecting the energy levels and transition dipole moments, which can enhance or modify the efficiency of energy transfer compared to classical predictions.^[35]^ Förster's model quantitatively incorporates the distance dependency of the dipole‐dipole interaction, taking into account the overlap integral, the quantum yield of the acceptor, the lifetime of the donor in the absence of an acceptor, and the effective index of refraction of the medium. The distance was such that the rate of energy transfer from the donor to the acceptor was comparable to the rate of fluorescence emission from the donor, indicating a significant efficiency of energy transfer.

**Figure 2:**
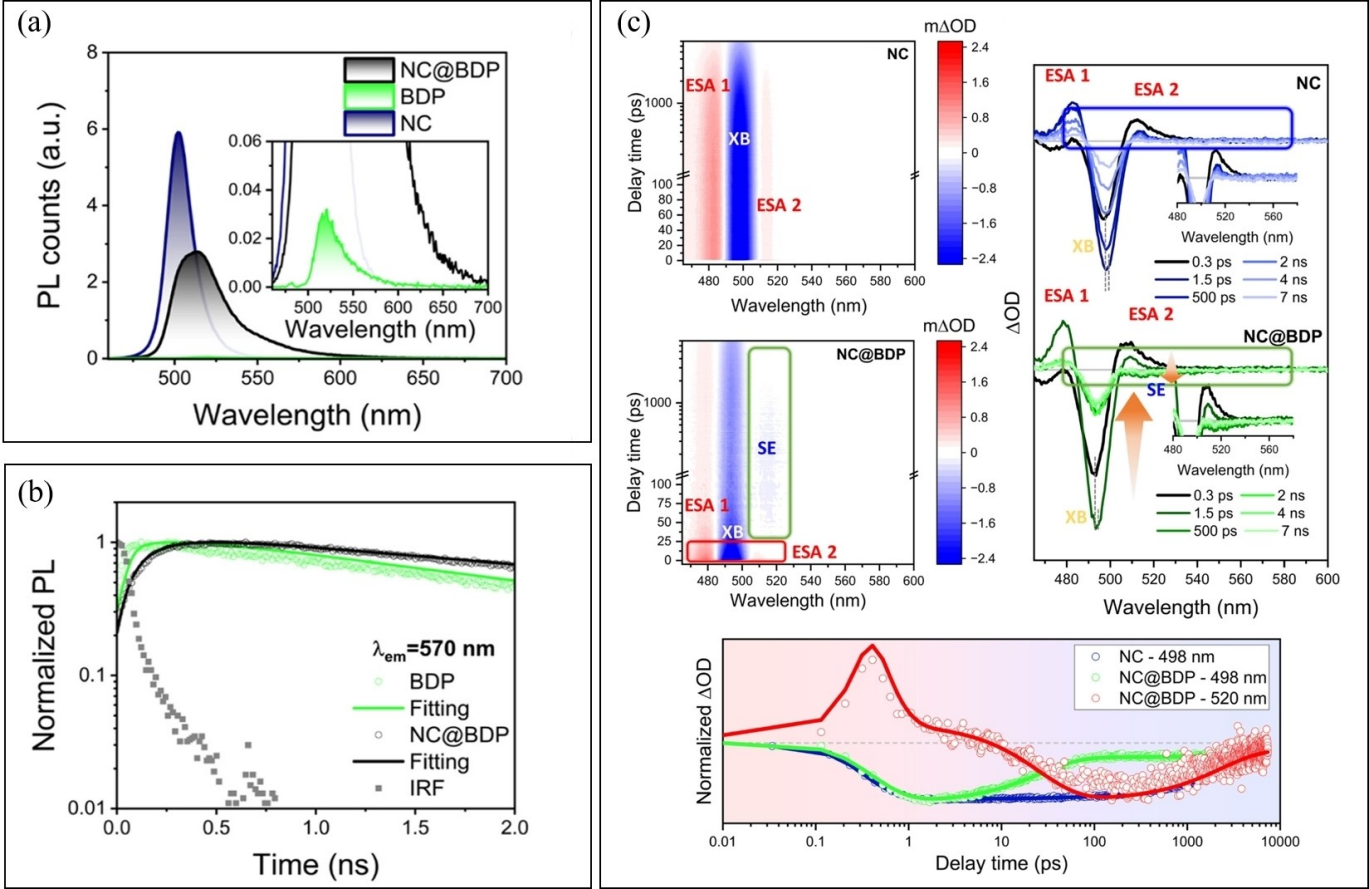
a) The PL spectra of perovskite NCs, organic dopant and perovskite‐organic nanohybrids (inset enlarged emission). b) PL decay traces of perovskite NCs, organic dopant and perovskite‐organic nanohybrids, where organic dopant was emissive. c) Time‐resolved transient absorption data of perovskite NCs and perovskite‐organic nanohybrids in toluene using a 420 nm pump.^[36]^ Copyright 2024 American Chemical Society.

There have several techniques to characterize the FRET process: Firstly, it is necessary use the steady‐state absorption and photoluminescence (PL) spectrometry to check the spectral overlap of the absorption of the dopant and the PL of the host. As shown in Figure 1(c), FRET occurs only with the overlap of the absorption and PL spectra. Once the condition of overlap is satisfied, time‐resolved photoluminescence (TRPL) and the femtosecond transient absorption spectroscopy (fs‐TAS) can be used to investigate the ultrafast energy transfer process. For instance, the fluorescence decay of dopant from the TRPL can reveal the sensitization process with the increasement of PL lifetime of dopant compared with the pristine system (Figure 2(a) and (b)). The ultrafast dynamics of the hot‐ and band‐edge excitons and the evolution of charge carriers could be observed from the TAS measurement (Figure 2(c)).^[36]^

## 3 Förster Resonance Energy Transfer in Metal Halide Perovskites

MHPs are emerging optoelectronic materials widely used in photovoltaics, LEDs, photodetectors, etc., they often exist in complicated systems including perovskite‐perovskite, perovskite‐inorganic quantum dots (QDs), perovskite‐organic fluorophores, or organic fluorophores‐perovskite. Energy transfer can proceed within these systems allows for efficient manipulation of electrons/excitons behavior in the excited states.

### 3.1 Perovskite‐Perovskite

Perovskite nanocrystals (NCs) exhibit strong, narrow and tunable emission in the wavelength from 410 to 790 nm along with near unity photoluminescence quantum yield (PLQY) without intricate surface passivation These excellent optical properties are closely related to the electronic structure of perovskite NCs, which lead perovskite NC to serve as the donor in non‐radiation energy transfer process. The excellent and facile optical properties are related to the electronic structure of perovskite NC, which lead perovskite NC to serve as the donor in non‐radiation energy transfer process.

**Figure 3:**
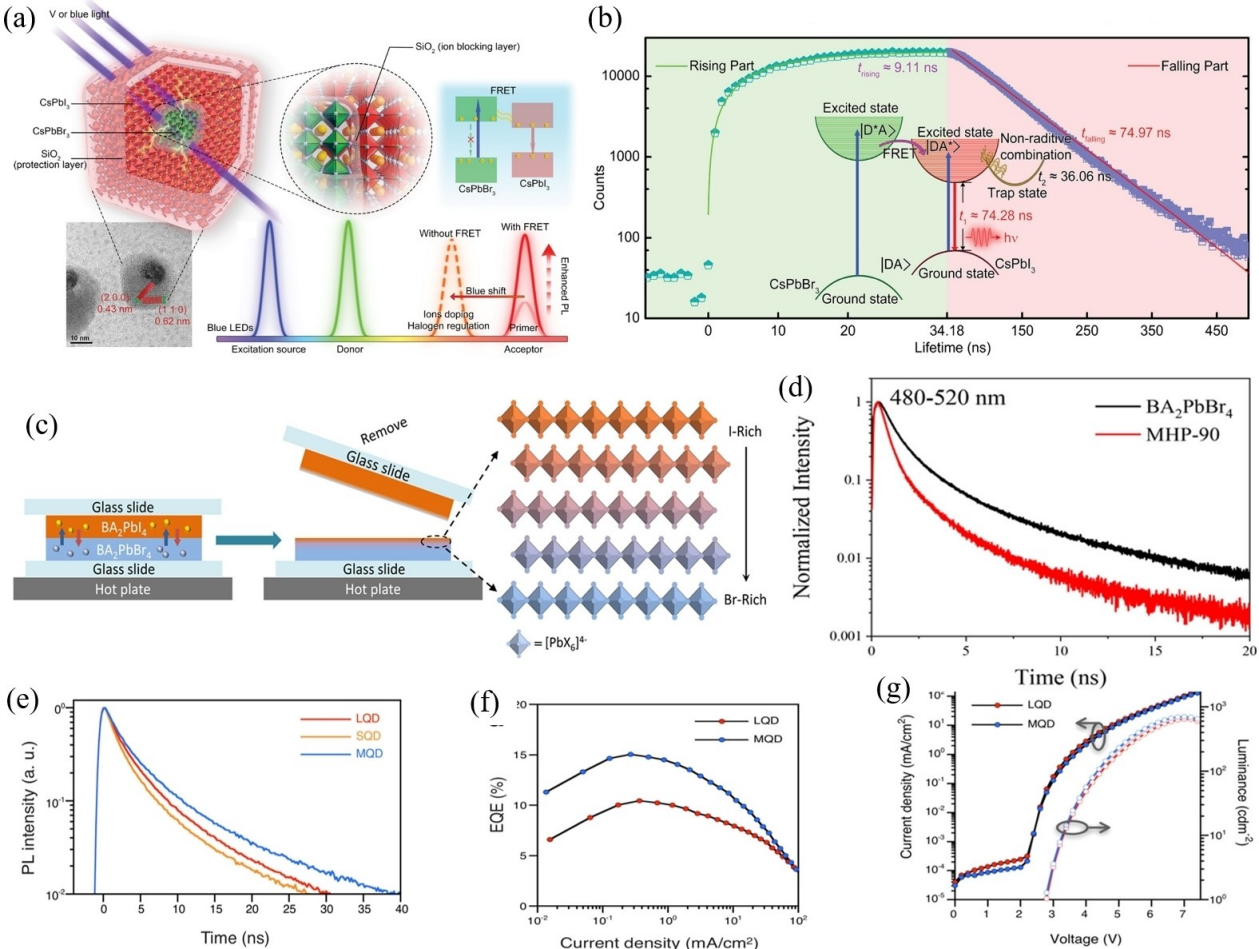
a) The bicomponent core–shell multilayer structure, TEM image, PL, b) TRPL of BPNC. diagram of enhanced PL of BPNCs. c) Schematic of the preparation process of the mixed halide perovskite crystal. d) The TRPL of the BA2PbBr4 perovskites and MGP‐90. e) PL decay of the large‐QD, small‐QD, and mixed‐QD with small‐QD/large‐QD=4 : 6 v/v in film. f) The external quantum efficiency–current density characteristics of PeQD LED. j) The current density–luminance–voltage characteristics of the LED. (a,b) Adapted with permission from ref. [37] Copyright 2023 Wiley‐VCH GmbH. (c,d) Adapted with permission from ref. [38] Copyright 2023 American Chemical Society. (e,f,g) Adapted with permission from ref. [39] Copyright 2022 American Chemical Society.

In the perovskite‐perovskite interaction, the core/shell strategy is utilized to shorten the distance between perovskite donor and perovskite acceptor. A CsPbBr_3_ core‐CsPbI_3_ shell structure is constructed. The green‐emitting CsPbBr_3_ in the core acts as the donor and the red‐emitting CsPbI_3_ in the shell acts as the acceptor. Efficient FRET is achieved through a pair of combined quasi‐degenerate energy levels in the blue spectra region.^[37]^ Owing to the highly efficient energy transition route from blue excitation to red emission established by the FRET, the core/shell perovskite nanocomposite exhibits the brightest single‐peak red photoluminescence with near 100 % quantum yield (Figure 3(a) and (b)). Because of the rich facility of the perovskite with different bandgaps. FRET is also possible when the perovskite donor and perovskite acceptor with different perovskite induced by the gradient halide distribution. For example, FRET has been found in a vertical gradient band‐gap heterostructures of two‐dimensional (2D) layered perovskites of BA_2_PbBr_4_ and BA_2_PbI_4_ (Figure 3(c)).^[38]^ The time‐resolved photoluminescence (TRPL) measurement proved the faster carrier transfer of the diffused mixed halide crystal, which could be a result of the gradient band gap led by the FRET effect (Figure 3(d)). The mixed halide sample had an enhanced carrier transport due to the FRET effect and was confirmed to be a highly stable and phase segregation‐free material. In addition to the halide composition induced FRET between perovskites with different halide distribution, the exploration of geometry control of the perovskite quantum‐confined nanocrystals can also provide way for FRET (Figure 3(e)). In the two‐dimensional CsPbBr_3_‐based nanoplatelets system, precisely controlling the thickness of CsPbBr_3_ nanoplatelets has been reported to induce we observe an enhanced efficient FRET, along with corresponding transfer rates up to *k_FRET_* =0.99 ns^−1^ and efficiencies of nearly *η_FRET_* =70 %. Furthermore, FRET from small perovskite quantum dots (PeQDs) to enhance the PLQY of the film and LED efficiency (Figure 3(f) and (g)).^[39]^

### 3.2 Perovskite‐Inorganic Fluorophores

FRET between the MHP nanocrystals and CdSe/ZnS quantum dots (QDs) has been studied in a blend structure.^[40]^ The FRET efficiency of CsPbBr_3_PNCs with CdSe/ZnS QDs is greatly affected when anion‐exchanged to CsPbCl_3_. A drop in the FRET efficiency from 85 % (CsPbBr_3_) to 5 % (CsPbCl_3_) with QDs was observed.

**Figure 4:**
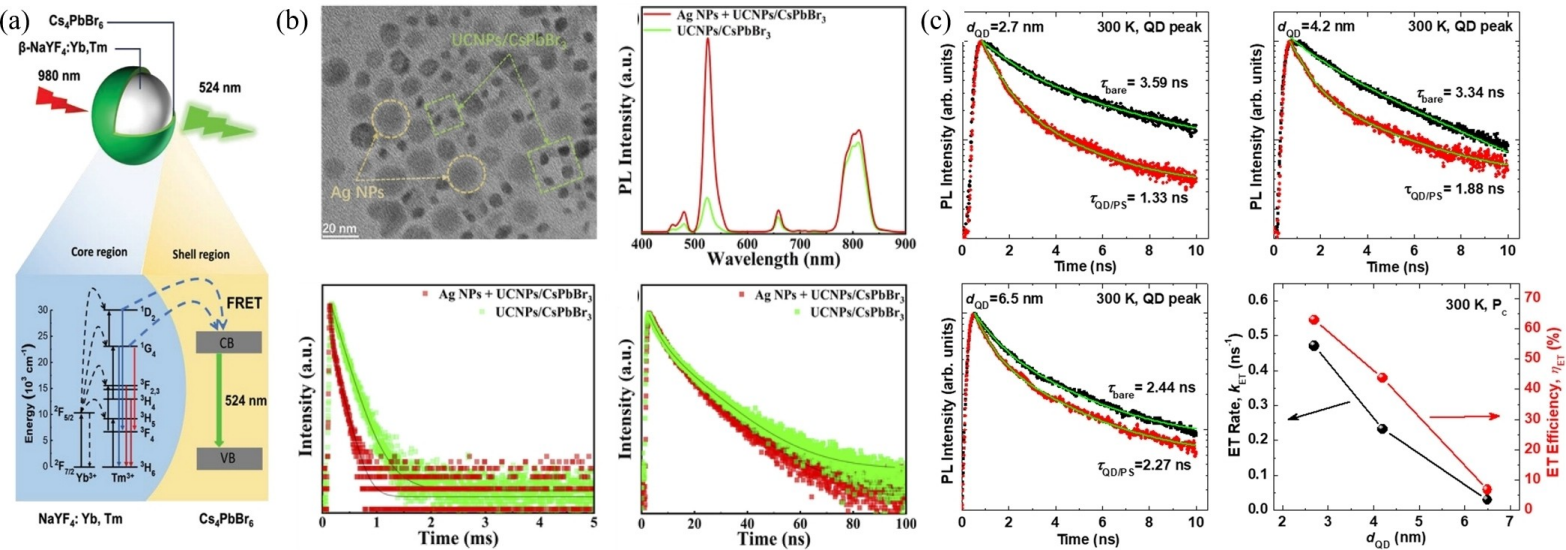
a) Schematic illustration of *β*‐NaYF_4_:Yb,Tm/Cs_4_PbBr_6_ core/shell NCs and the FRET process from Tm^3+^ to the conduction band of Cs_4_PbBr_6_. b) TEM image, PL spectra, Time‐resolved decay curves monitored at 481 nm and 526 nm of UCNPs/CsPbBr_3_ nanocomposites with Ag NPs. c) PL decay curves at QD peak for the bare QDs (black dots) and QD/PS hybrid structures (red dots) with QD of 2.7 nm, 4.2 nm, and 6.5 nm. (a) Adapted with permission from ref. [41] Copyright 2023 Wiley‐VCH GmbH. (b) Adapted with permission from ref. [42] (c) Adapted with permission from ref. [43] Copyright The Author(s) 2019 SCIENTIFIC REPORTS.

The donor‐acceptor system is a good choice for improve the performance of perovskite materials With the heteroepitaxial growth of Cs_4_PbBr_6_ on the surface of ***β***‐NaYF_4_ to form a high‐quality ***β***‐NaYF_4_:Yb,Tm/Cs_4_PbBr_6_ core/shell nanocrystal structure (Figure 4(a)), the FRET efficiency was able to largely improved up to 58.33 %, compared with a physically mixed sample of 1.84 %.^[41]^ The core/shell NCs emitted narrow‐band green light emission attribute to the FRET from Tm^3+^ to Cs_4_PbBr_6_. A similar work on the up‐conversion energy transfer between inorganic nanoparticles (UCNP)of BaYF_5_: Yb, Tm to CsPbBr_3_ PeQDs was also demonstrated (Figure 4(b)).^[42]^ The nanocomposites had enhanced sixfold on the green up‐conversion luminescence at 526 nm with the FRET efficiency of approximately 31.1 %. CdSe/ZnS QDs also showed the ability to transfer charge or exciton to perovskite.^[43]^ In the QDs passivated Perovskite film, the FRET efficiency was found to rely on the core diameter of CdSe/ZnS QDs, which was increased from 7 % to 63 % when the core diameter decreased from 6.5 to 2.7 nm (Figure 4(c)). the enhanced energy transfer efficiency was explained by the charge transfer rate enhancement due to the control of energy level alignment of CdSe/ZnS QDs.

### 3.3 Perovskite‐Organic Fluorophores

**Figure 5:**
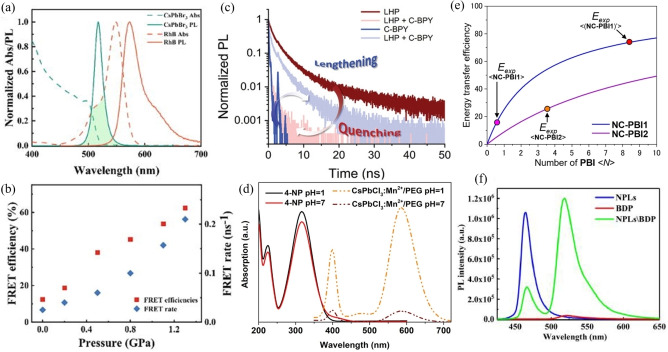
a) Normalized absorption and PL spectra of RhB molecules and CsPbBr_3_ QDs. b) Correlation between pressure change, FRET rate, and FRET efficiency. c) Normalized NCs and C‐BPY life time decay traces were recorded at 502 and 650 nm, respectively. d) UV–visible absorption spectrum of 4‐NP and the PL spectrum of CsPbCl_3_/Mn^2+^/PEG NCs and at pH=1 and 7 (λ_ex_=250 nm). e) Correlation curves between theoretical fluorescence resonance energy transfer (FRET) efficiency and the number of acceptors. f) PL spectra of BODIPY, CsPbBr_3_ NPLs and their complexes under excitation at 420 nm. (a,b) Adapted with permission from ref. [44] Copyright 2023 Wiley‐VCH GmbH. (c) Adapted with permission from ref. [45] Copyright 2023 AIP Publishing. (d) Adapted with permission from ref. [46] Copyright 2024AmericanChemicalSociety (e) Adapted with permission from ref. [47] Copyright 2024 American Chemical Society (f) Adapted with permission from ref. [48] Copyright 2024 Optics Express.

MHPs are excellent donor materials in the perovskite‐organic system. The widely studied FRET process in the perovskite‐organic fluorophores system is between perovskite‐ Rhodamine B (RhB).^[44]^ With pressure regulation, the FRET rate of the CsPbBr_3_‐PhB composite reached 0.21 ns^−1^ and the FRET efficiency is improved from 12.4 % to 62.4 %, due to enhanced spectral overlap and shortened minimum distance between CsPbBr_3_ QDs and RhB molecules (Figure 5(a) and (b)). In perovskite nanocrystals, its surface is often covered with organic ligands. The surface organic ligands play key role in passivate the defects of perovskite nanocrystals and the mediate the FRET between MHPs and organic molecules.^[45]^ FRET efficiency was up to 85 % with the strong binding of the organic molecule to the MHP NC surface via the carboxylate group was demonstrated (Figure 5(c)). In order to make PeQDs suitable in biochemical detection, a strategy to was developed to encapsulate Mn^2+^‐doped CsPbCl_3_ PeQDs within poly(ethylene glycol) (PEG) to effectively increases the water stability of PeQDs.^[46]^ The CsPbCl_3_/Mn^2+^/PEG NCs had an interaction with 4‐Nitrophenol (4‐NP) to establish a fast and conservative fluorescent probe, which depended on the FRET between CsPbCl_3_/Mn^2+^/PEG NCs (donor) and 4‐NP (acceptor) (Figure 5(d)). The coating pf perovskite nanocrystals with self‐assembly dye such as perylene bisimide (PBI) derivatives also led to FRET from perovskite nanocrystals to the monomeric PBIs, because of the reduce distance between the perovskite donor and the PBI acceptor.^[47]^ This self‐assembly of PBI on the NC surface had efficient reduce the distance of the donor (NC) and acceptor (PBI) (Figure 5(e)). Not only PBI derivatives and perovskite NCs can proceed FRET, but also happened between boron dipyrromethene (BODIPY) and perovskite nanoplatelets (CsPbBr_3_ NPLs).^[48]^ The quenching of PL in CsPbBr_3_ NPLs occurred simultaneously with the PL enhancement of BODIPY implied the singlet energy transfer process. The efficiency of FRET has been quantitatively calculated up to 70 % (Figure 5(f)).

### 3.4 Organic Fluorophores‐Perovskite

**Figure 6:**
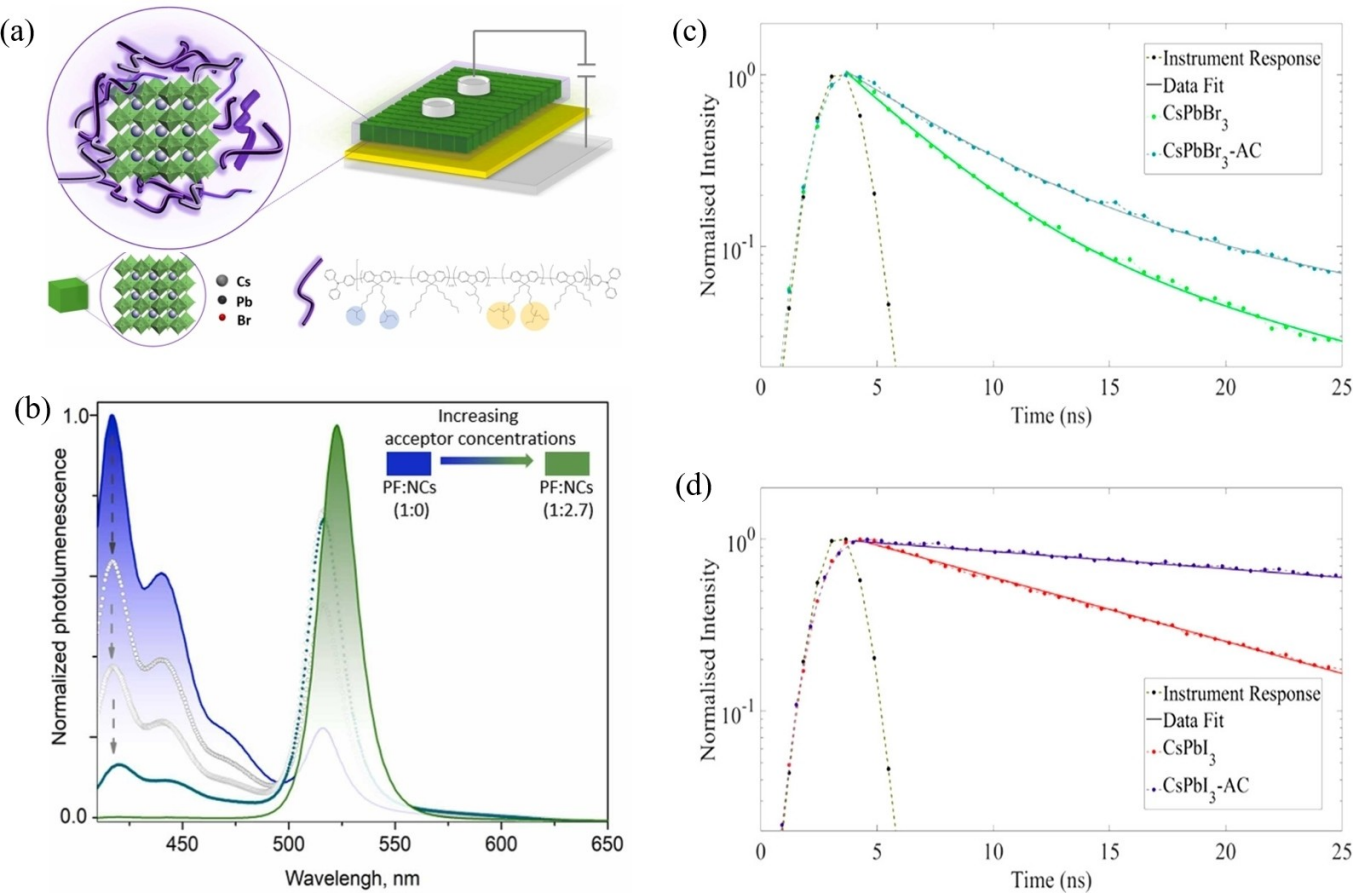
a) A schematic illustration of the interaction between PF polymers with perovskite NCs surface and the schematic structure of hybrid composite LEDs. b) PL spectrum of PFs and the absorption spectrum of CsPbBr_3_ NCs. c) PL Decay overlay of AC, oleate capped CsPbBr_3_, and CsPbBr_3_‐AC. d) PL Decay overlay of AC, oleate capped CsPbI_3_, and CsPbI_3_‐AC. (a,b) Adapted with permission from ref. [49] Copyright 2024 Elsevier B.V. (c,d) Adapted with permission from ref. [50] Copyright 2021 American Chemical Society.

The research on energy transfer from organic host to perovskite is sparse. There has been energy transfer channel from the band of the inorganic system to the singlet or triplet oof organic fluorophores. Singlet and triplet energy transfer have been reported between perovskite NCs and organic dyes. Although the characteristics of the absorption and emission spectra of perovskite and organic fluorophores make perovskite to be more likely as donor and organic material as acceptor, researchers tried to realize the FRET process base on organic material as the donor and perovskite as the acceptor. This approach is promising as perovskite nanocrystals still undergo the problems of surface defects and aggregation. The organic donor could be a solution to address these issues to enhance light emission, as the functional groups, such as amino group and triphenylphosphine group, can be decorated on the organic donor to passivate the halide vacancies or the uncoordinated Pb^2+^ defects. Besides, the organic host can form a core‐shell structure with QDs, which can protect the perovskite QDs from aggregation. For example, *A.V. Yakimansky* developed a class of modular polyfluorene (PF) copolymers, which designed to stabilize perovskite NCs (Figure 6(a)).^[49]^ Interestingly, the polymer/NCs composite showed efficient FRET from PF to NC with green PL (Figure 6(b)). The PF host not only strengthen the light emitting in solid state, but also provide an efficient charge transfer to NC emitter in LED. Furthermore, FRET has demonstrated its potential to enhance the light energy utilization ratio of perovskite solar cells by interacting with metal‐organic frame works (MOFs) and perovskite layers. *J. L. K. Davis* demonstrated a direct in situ attachment of anthracene‐9‐carboxylic acid ligands to the surface of CsPbBr_3_ and CsPbI_3_ NCs. FRET process from the anthracene ligands with efficiencies as high as 40 % was demonstrated (Figure 6(c) and (d)).^[50]^

In our assumption, a possible way of the energy transfer from the organic host to and perovskite dopant can be realized through the organic ligands of perovskite NCs, whereas the organic ligands worked as the intermediate media for the transfer process. The intermediate media should share the same molecular system with organic host. moreover, the organic host and organic ligand can construct a charge‐transfer system.

## 4 Future Outlooks

The utilization of FRET in MHPs has attracted significant interest due to its potential to improve device efficiency, enable color adjustments, and introduce advanced functionalities. This process allows for energy transfer among different components based on halide perovskite. For example, FRET from high bandgap perovskite donor to low bandgap acceptor materials can reduce non‐radiative recombination pathways, leading to improved charge carrier extraction and device performance. Another advantageous aspect of FRET in MHPs is its capability to facilitate precise color adjustments and spectral engineering in optoelectronic devices. By carefully selecting donor and acceptor materials with distinct emission spectra, researchers can control energy transfer and manipulate the emission properties of perovskite‐based devices. These potentials open doors for the creation of customizable light sources, displays, and sensors with tailored emission wavelengths and color ranges to meet diverse application needs. Moreover, FRET‐based sensing platforms can utilize the sensitivity of energy transfer processes to detect variations in environmental conditions like temperature, humidity, or chemical analyte concentrations. In addition, the integration of FRET into perovskite‐based photonic and optoelectronic circuits has the potential to catalyze the enhancement of advanced devices for information processing, communication, and sensing purposes. For instance, the FRET mechanism can be adopted to build an organic host‐perovskite dopant system as color conversion layer for full‐color micro‐LED display, as the organic host can be designed with strong blue light absorption, transfer the energy to perovskite dopant through the FRET process, and emit high color purity green and red light for color conversion display. The bioimaging can have high‐resolution image using FRET from the biological tissues or cells to the perovskite, with high PLQY, narrow emission, and nonlinear optical properties. While the potential for FRET in halide perovskites is optimistic, there exist several challenges that require attention, including the optimization of FRET efficiency, enhancement of material stability, and development of scalable fabrication techniques for FRET‐based devices. It is crucial to have a fundamental understanding of FRET mechanisms in MHPs and how they interact with device structures in order to fully realize their potential. Handling these challenges opens doors for interdisciplinary research collaboration and technological advancements in the area of halide perovskite optoelectronics.

## 5 Summary

In conclusion, FRET has considerable potential to enhance the performance, functionality, and adaptability of optoelectronic devices based on MHPs. The researchers can improve the efficiency of the device, obtain precise spectral control, and enable unique functionality in a wide variety of applications by utilizing the principles of energy transfer. As research in this area continues to advance, the incorporation of FRET into halide perovskite technologies is expected to change the landscape of optoelectronics. Efficient energy transfer among different components in devices based on halide perovskites is enabled by the process of FRET, which can lead to improved charge carrier extraction and device performance.

## Conflict of Interests

The authors declare no conflict of interest.

6

## Data Availability

The data that support the findings of this study are available from the corresponding author upon reasonable request.
